# Splicing genes as main targets of differential splicing

**DOI:** 10.1093/g3journal/jkag213

**Published:** 2026-08-14

**Authors:** Torstein Tengs, Thomas LaFramboise, Yossef Baidi, Rameen Beroukhim, Won-Young Choi, Hyo Young Choi, David Neil Hayes

**Affiliations:** Department of Breeding and Genetics, Norwegian Institute of Food, Fisheries and Aquaculture (Nofima), Ås 1433, Akershus, Norway; Department of Genetics and Genome Sciences, Case Western Reserve University, Cleveland, OH 44106, United States; Broad Institute of Harvard and MIT, Cambridge, MA 02142, United States; Department of Medicine, Harvard Medical School, Harvard University, Boston, MA 02215, United States; Department of Medicine, Harvard Medical School, Harvard University, Boston, MA 02215, United States; Department of Cancer Biology, Dana-Farber Cancer Institute, Boston, MA 02215, United States; Department of Medicine, The University of Tennessee Health Science Center, Memphis, TN 38163, United States; UTHSC Center for Cancer Research, The University of Tennessee Health Science Center, Memphis, TN 38163, United States; Department of Computational Biology, St. Jude Children's Research Hospital, Memphis, TN 38105, United States; Department of Medicine, The University of Tennessee Health Science Center, Memphis, TN 38163, United States; UTHSC Center for Cancer Research, The University of Tennessee Health Science Center, Memphis, TN 38163, United States; Department of Preventive Medicine, The University of Tennessee Health Science Center, Memphis, TN 38163, United States; Department of Medicine, The University of Tennessee Health Science Center, Memphis, TN 38163, United States; UTHSC Center for Cancer Research, The University of Tennessee Health Science Center, Memphis, TN 38163, United States; Department of Computational Biology, St. Jude Children's Research Hospital, Memphis, TN 38105, United States

**Keywords:** splicing, mRNA, spliceosome, Animalia

## Abstract

Splicing of transcripts via the spliceosome machinery is a complex process involving a multitude of proteins and short noncoding RNAs. In addition to full-length transcripts with all exons in the genomically encoded order, alternatively spliced transcripts can also be produced through alternative splicing of pre-mRNA. We have attempted to identify which genes are most frequently targeted by differential splicing using both downloaded and original RNA-Seq data from several species and tissue types. In order to identify splicing variation on an individual-to-individual basis, random contrasting of samples within compatible sets of samples was done. Both when analyzing sequence data from similar source material (same tissue from individuals kept under similar conditions) and when contrasting samples that are more different, such as different tissue types, genes associated with the splicing process itself seem to be the most recurring targets.

## Introduction

Splicing of mRNA is a basic biological mechanism that enables the production of a multitude of transcripts from the same gene locus. In eukaryotes, the spliceosome, a large ribonucleoprotein complex, is largely responsible for the precise cutting and re-ligation of transcripts, where intronic regions are removed and coding regions joined to produce a coding sequence. The spliceosome appears to be highly dynamic, but there are evolutionary conserved features (Kastner et al. 2019). In addition to the core spliceosome, a collection of associated RNA splicing proteins, heterogeneous nuclear ribonucleoproteins, and other components are necessary for correct splicing to occur. Small nuclear ribonucleoproteins are also essential constituents of the spliceosome, and through their interactions with other elements, it is believed that these complexes are key in defining pre-mRNA exonic and intronic regions (Ule and Blencowe 2019).

In addition to full-length transcripts containing a complete set of exons in the predicted order, splicing can also produce restructured coding sequences through alternative splicing (Marasco and Kornblihtt 2023). In the human genome, it has been estimated that approximately 95 % of multiexon genes exhibit alternative splicing (Pan et al. 2008), and alternative splicing has been linked to a wide range of biological processes, including tissue identity (Baralle and Giudice 2017), infrared sensing (Gracheva et al. 2011), glycolysis (Choksi et al. 2021), muscle adaptation (Ubaida-Mohien et al. 2019a, 2019b), and loss of tail in hominoids (Xia et al. 2024). Aberrant splicing has also been linked with diseases, such as autism (Irimia et al. 2014) and cancer (Bradley and Anczukow 2023). In a set of tumors from different cancer types, 68 % were found to contain alternative splicing-derived neoepitopes (Frankiw et al. 2019), and tumors have been reported to have up to 30 % more alternative splicing than normal tissue (Kahles et al. 2018).

We have attempted to establish which genes most commonly show alternative splicing variation, and if these genes are linked to specific processes. We have focused our analyses on RNA-Seq data from 3 different sources: nonhuman model organisms, data from healthy humans, and data from different human tumor types. Sample sets range from highly homologous tissues (i.e., liver samples from zebrafish kept under identical conditions) to data derived from different types of human tissues, including the liver, lung, spleen, adipose tissue, pancreas, thyroid gland, and kidney. As splicing dynamics has also been implemented in cancer development, sets of tumors from different cancer types and their matching normal adjacent tissue samples were also analyzed.

Splice variant differences were assessed by using random contrasting of samples and detection of local splicing variation (LSV). All analyses point to the components of the spliceosomal machinery as being the most frequent target of alternative splicing, a trend that was pronounced even when contrasting samples of very similar origin.

## Materials and methods

### Zebrafish RNA-Seq

In order to investigate any differences in splicing patterns in highly homologous samples, we sought to generate data from a model organism that could be kept under as stable conditions as possible, and chose a tissue known to have high transcriptional activity. Zebrafish (*Danio rerio*, strain ABwt) were raised at The Model Fish Unit (Norwegian University of Life Sciences, Faculty of Veterinary Medicine, Ås, Norway). The fish were housed in an Active Blue standalone rack system (Tecniplast, Buguggiate VA, Italy) and raised on a diet of dry feed (Zebrafeed; Sparos, Olhão, Portugal) morning and afternoon, and a*rtemia* (Sep-Art a*rtemia*; Ocean Nutrition, Dartmouth, Canada) at lunchtime. The fish were kept at a density of 20 fish per 3.5 L. The light regime was 14:10 (light:dark) with 30 min of dawn and dusk transitions. Water temperature was kept at 27.5 °C, average pH was 7.7, and conductivity was 750 µS. Ammonia, nitrite, and nitrate were monitored weekly and kept at low levels by daily water exchange of 15 % to 25 %.

Liver tissue was dissected from 10 adult fish, and RNA extracted using the RNAdvance Tissue kit (Beckman Coulter, Indianapolis, IN, USA). RNA was prepared for 150-bp paired-end Illumina RNA-Seq using the TruSeq Stranded mRNA kit and the NovaSeq X platform (Illumina, San Diego, CA, USA). Sequencing was done at the Norwegian Sequencing Centre (Department of Medical Genetics, Oslo University Hospital, Oslo, Norway).

Reads were mapped to the zebrafish genome using the STAR RNA-Seq aligner (Dobin et al. 2013) (version 2.7.3a) with default parameters. Sequences and annotation files were downloaded from the Ensembl database (Harrison et al. 2024) (Danio_rerio.GRCz11; Supplementary Table 1). bam files produced were sorted and indexed using samtools (Danecek et al. 2021) (version 1.10) with HTSlib version 1.10 and subsequently analyzed using the MAJIQ pipeline (Vaquero-Garcia et al. 2023) (version 2.5.7). First, MAJIQ build was run in order to define known and novel splice variants. Then, all possible contrasts between the individual fish (*n* = 45) were analyzed using the MAJIQ deltapsi module to calculate differences in splice variant populations between individuals. The output from deltapsi was processed further using MAJIQ modulizer, and genes that were scored as showing significant splicing differences (default MAJIQ cutoff values; dpsi_changing_threshold: 0.2, dpsi_nonchanging_threshold: 0.95, dpsi_probability_changing_threshold: 0.95) in a contrast were tabulated. In order to reduce a bias toward long/complex genes, a gene was only counted as being differentially spliced once per contrast. Using the modulizer output files, the frequency of a gene being differentially spliced was calculated, and genes were ranked.

### rMATS turbo analysis

In addition to the MAJIQ pipeline, our original zebrafish data were also contrasted and analyzed using the rMATS turbo pipeline (Wang et al. 2024). The default rMATS turbo STAR RNA-Seq aligner was used as described above for MAJIQ, combined with rMATS turbo version 4.3.0 (default parameters), and the same random contrasting of samples was also done. The output was filtered to include only events with false discovery rate < 0.05, and further processing was done as described for the MAJIQ data.

### Additional species and human data

Paired-end RNA-Seq data from other species and tissues were downloaded from the National Center for Biotechnology Information (NCBI) Sequence Read Archive (SRA). For nonhuman samples, data were downloaded from yeast (*Saccharomyces cerevisiae*) and *Caenorhabditis elegans* (RNA extracted from whole organism), as well as different fruit fly (*Drosophila melanogaster*) body segments and mouse (*Mus musculus*) livers. In addition, a zebrafish liver dataset (Leonard et al. 2023) similar to our original data (Supplementary Table 1) was also included.

Data from many different human tissue types and sequences from studies on cancer transcriptomics were also downloaded from the SRA database (Supplementary Table 1). The majority of the normal human tissue data were from the ENCODE project (Consortium 2012), and here samples were combined to make sets of tissues with overlapping description (“cerebellum” was combined with “brain,” “suprapubic skin” was combined with “skin,” etc.; Supplementary Table 1).

Annotation files for different genomes/transcriptomes were downloaded using Bioconductor (Gentleman et al. 2004) (release 3.20), and data were analyzed as described above for zebrafish, with the reads aligned to the latest version of the respective species’ genomes (Ensembl data (Harrison et al. 2024); Supplementary Table 1). For the human data, samples where <80 % of the reads could be uniquely mapped were excluded. Within each sample set, random contrasts were performed using all possible pairs of samples, with an upper limit of 300 permutations (Supplementary Table 1).

For all the sample sets, random contrasting was performed as described above for zebrafish. For yeast, the sample set contained sequences from 50 samples, allowing for >300 permutations. The mouse liver dataset contained 35 samples, also permitting >300 random contrasts. For the other nonhuman samples, variable numbers of permutations were possible given the number of samples in the different sets (*C. elegans*, 91; fruit fly carcass, 66; fruit fly hindgut, 36; and fruit fly midgut, 36 contrasts; Supplementary Table 1). For the human normal tissue sample sets, 35 RNA-Seq run collections were downloaded, representing 31 different tissue types and a number of possible random contrasts ranging from >300 to 3 (Supplementary Table 1). In the cancer-associated analysis, 8 different sets of tumor samples were analyzed, 2 of which could also be contrasted with matched normal tissue (Supplementary Table 1).

### clusterProfiler analyses

The 20 % highest-ranked genes (most frequently differentially spliced) from the MAJIQ analysis were used as input in enrichment analyses using the R package clusterProfiler (Xu et al. 2024) (version 4.14.3). Analyses were done using the Gene Ontology (GO; Ashburner et al. 2000; Gene Ontology et al. 2023) “Biological Process” and “Cellular Compartment” (enrichGO), Kyoto Encyclopedia of Genes and Genomes (KEGG) (Kanehisa et al. 2025) (enrichKEGG), and WikiPathways (Agrawal et al. 2024) (enrichWP) gene sets.

For the nonhuman samples, all annotated genes were used as the background universe, whereas for the human samples, genes were ranked using the raw STAR counts data, and the 5,000 genes with the highest level of expression were used (average counts from all human samples) to avoid read count bias. For each of the 4 different annotation sources, the top 10 enriched terms were extracted, and terms with an adjusted *P* > 0.05 were removed.

### Gene Set Enrichment Analysis

Complete lists of genes that showed differential splicing from analyses of the human ENCODE data were also used as input for Gene Set Enrichment Analysis (GSEA) (Mootha et al. 2003; Subramanian et al. 2005). GSEA is based on a ranking of genes using some relevant metric, and statistical tests are then done to check if there is an enrichment of genes belonging to a predefined set along an axis going from low-ranking to high-ranking genes. The analysis was done via the GenePattern platform (Reich et al. 2006) using the number of times a gene was scored as differentially spliced as the ranking metric. The GSEAPreranked module (version 7.4.0) was used with 100,000 permutations, and the REACTOME_MRNA_SPLICING human gene set from the Molecular Signatures Database was chosen to check for enrichment of genes involved in mRNA splicing (Reactome (Milacic et al. 2024) annotation).

## Results

### Zebrafish and other nonhuman data

For our original data derived from zebrafish liver tissues, an average of 92 million paired-end reads were generated per sample (average unique mapping rate of 64 %). The data were submitted to the NCBI SRA database as BioProject PRJNA1237738.

After aligning both our original zebrafish liver data and the downloaded datasets to their corresponding species’ reference genomes (Supplementary Table 1), sorted and indexed bam-files were analyzed using the MAJIQ pipeline. Lists of differential splicing events (MAJIQ summary.tsv output) were processed, and the number of events per gene was tabulated (Supplementary Table 2). The number of genes reported as having differentially spliced transcripts was highly variable after contrasting samples within the different sample sets, ranging from 65 in yeast to 1,922 in zebrafish (Supplementary Table 2). In addition to likely differences in the different species’ propensity for using differential splicing (Wright et al. 2022), these differences in number of events were most likely due to a combination of the number of samples included in the set (a high number of samples will allow for a high number random number of contrasts, which again will lead to a higher number of events being detected) and the number of genes in the different species’ genomes.

Top ranking terms from the enrichment analyses of the most frequently differentially spliced genes in the zebrafish were “RNA splicing” (GO:0008380; biological process GO term), “Spliceosome” (dre03040; KEGG), “mRNA processing” (WP467; WikiPathways), and “Nuclear body” (GO:0016604; cellular compartment GO term, parent to terms such as “Nuclear speck,” which includes spliceosomes (Lamond and Spector 2003)) (Fig. 1; Supplementary Table 3). These results were consistent with our analysis of a previously published zebrafish liver dataset (Leonard et al. 2023) (Fig. 1).

**Fig. 1.**
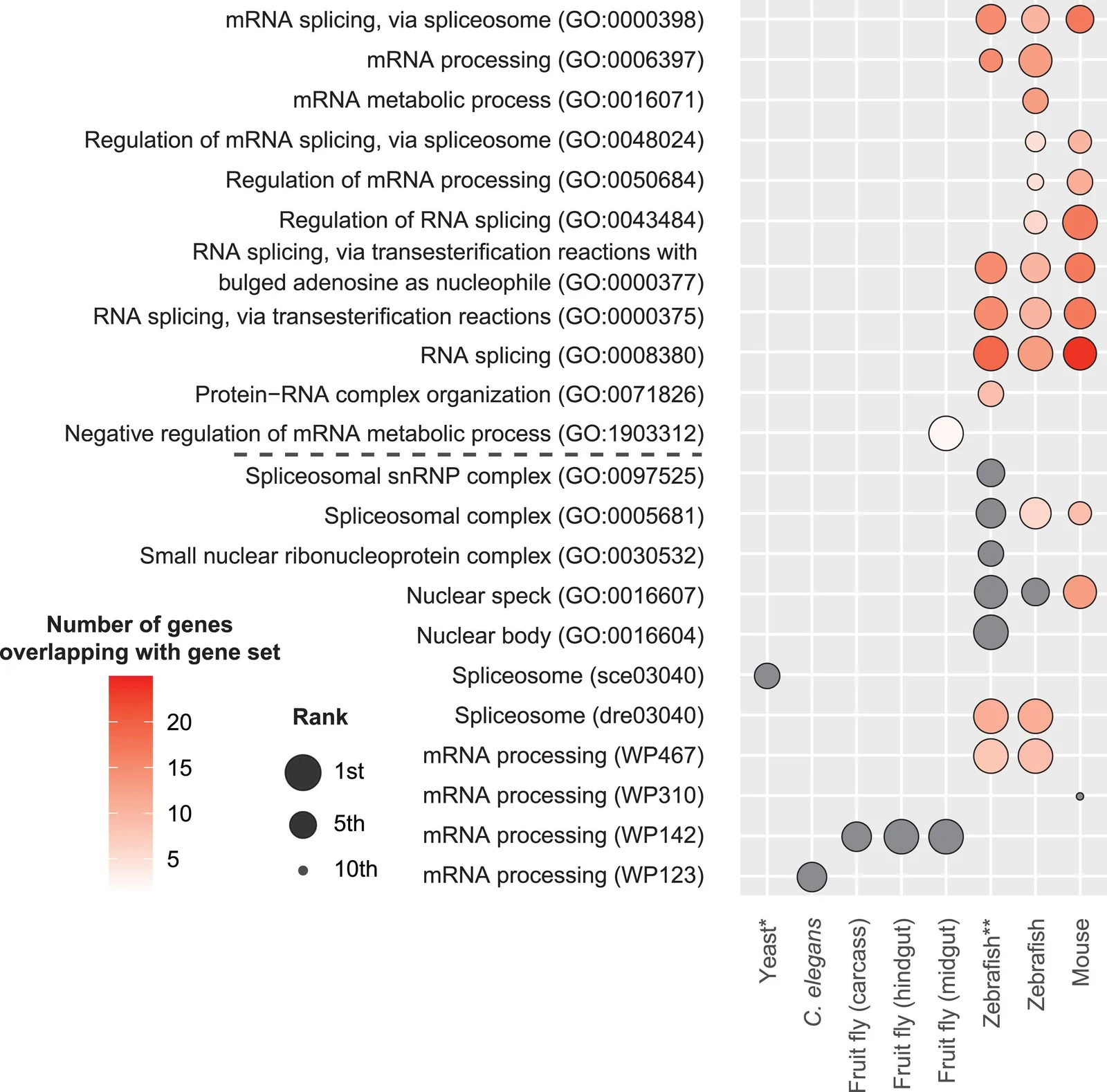
Enriched gene sets using the 20 % most frequently differentially spliced genes as input (nonhuman samples). Significant hits (within top 10 of ranked list, adjusted *P* < 0.05) relevant for splicing are shown, and the number of genes overlapping with the gene set indicates how many genes overlapped between the top 20 % genes and the gene sets reported as being significantly enriched. Above dotted line: biological process GO terms; below dotted line, cellular compartment GO terms, KEGG (“dre” and “sce”), and WikiPathways (“WP”) (gray fill; adjusted *P* > 0.05). *Due to the low number of genes that showed differential splicing in the yeast contrast (*n* = 65), the top 50 % was used as input for clusterProfiler. **Data from this study.

Differential splicing of genes linked with the splicing process was also observed in the downloaded mouse liver data as well as in the other species included (Fig. 1; Supplementary Table 3), but splicing term enrichment was not significant for *C. elegans* or yeast, and only marginally significant for a single GO term in the fruit fly data (“negative regulation of mRNA metabolic process”; adjusted *P* = 0.042; fruit fly midgut; Fig. 1). It has previously been reported that there is a lower overall number of splicing events in more primitive organisms (Wright et al. 2022). This was also a trend in our data, limiting the statistical power of our analysis.

When rMATS turbo was used to analyze our original data from zebrafish, very similar results were obtained, again showing a clear enrichment of terms associated with the splicing machinery (Supplementary Table 4).

### Human data

For the normal human tissues, the average number of differential splicing events reported was approximately 3 times higher in splicing-associated genes than other genes (Table 1), and an analysis of the human genome annotation file using GffRead (Pertea and Pertea 2020) (version 0.12.7) showed that per gene, an average of twice the number of isoforms has been reported from splicing genes compared with genes not associated with splicing (Table 1).

**Table 1.** Average number of differential splicing events reported per gene for genes where differential splicing was observed.

	Splicing-associated genes	Other genes
All genes	124.34	43.88
Highly expressed genes (top 5,000)	210.39	81.51
Lowly expressed genes (below top 5,000)	50.43	27.75
Average number of reported isoforms (all genes)^a^	12.12	6.72

Based on total set of contrasts (1,341) from normal human tissue ENCODE data.

^a^See Materials and methods for details.

The average number of genes that showed differential splicing in the analysis of normal human tissues was 2,457, many of which may represent a small fraction of the transcript pool for a given gene (Supplementary Table 2). The majority of the (sets of) differential splicing events were found more than once, including complex combinations (Supplementary Table 5). There was a significant enrichment of splicing-associated terms in the great majority of the normal human tissue types analyzed for all the different sources of gene annotation (Supplementary Table 3), with the exception of the brain, muscle, and umbilical cord (Fig. 2). For the cellular compartment GO terms, there was enrichment for “Nuclear speck” (GO:0016607), defined as “a discrete extranucleolar subnuclear domain, 20 to 50 in number, in which splicing factors (SFs) are seen to be localized by immunofluorescence microscopy” in the GO documentation, though the most commonly enriched compartment term was “Spliceosomal complex” (Fig. 2).

**Fig. 2.**
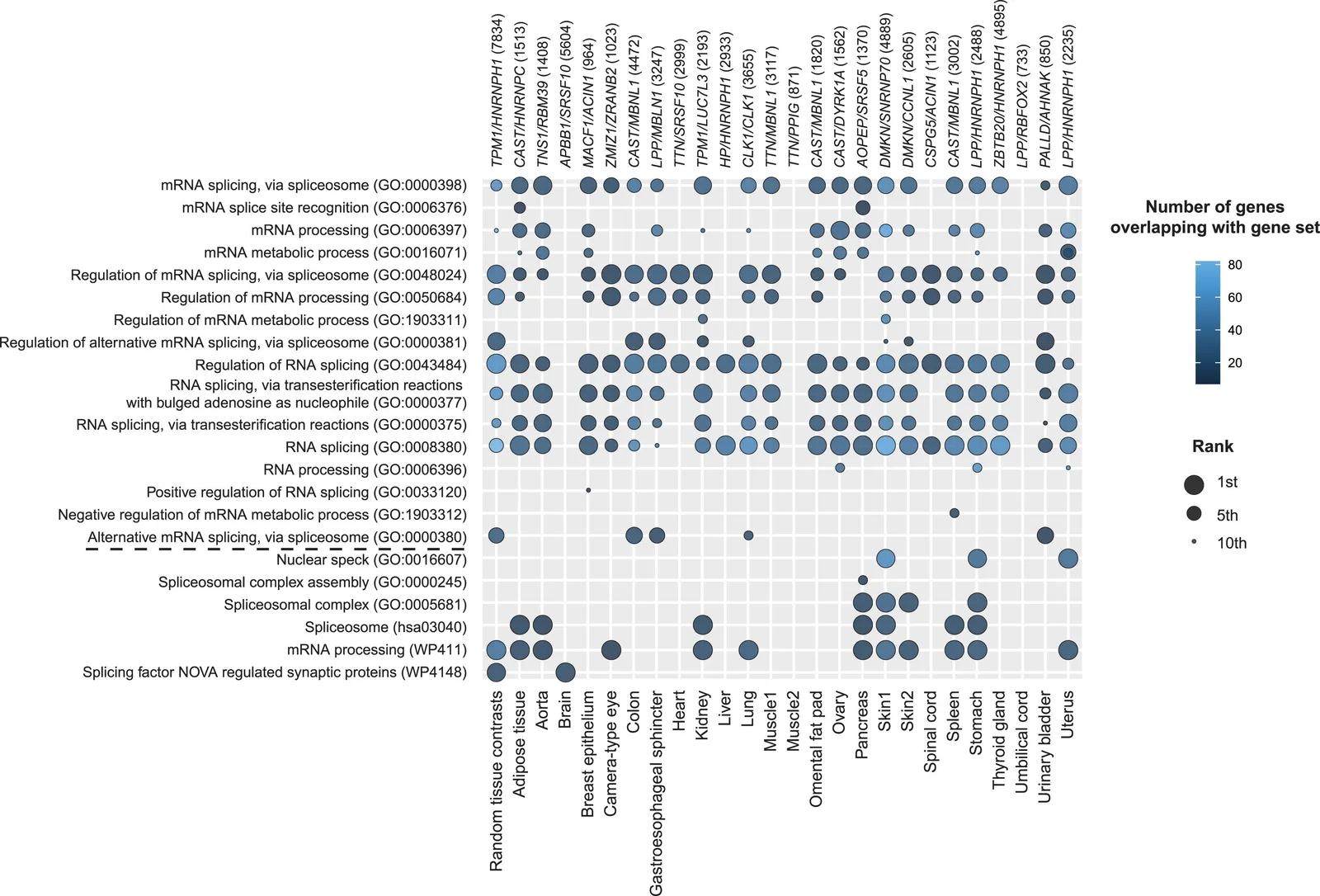
Enriched gene sets using the 20 % most frequently differentially spliced genes as input (normal human tissues). Genes most frequently reported as being differentially spliced are shown above the dot plot (all genes/spliceosome genes), in addition to the total number of genes reported as being differentially spliced in the contrasts. KEGG terms have prefix “hsa”; otherwise, annotations are the same as Fig. 1.

The GSEA analyses of normal human tissue samples showed a positive correlation between the frequency of differential splicing of a gene and its functional association with mRNA splicing (Supplementary Fig. 1). An analysis where all the different ENCODE datasets, representing different tissue types, were contrasted gave the highest normalized enrichment score (1.57; Fig. 3). All tissue types with a number of contrasts >50 gave *q*-values < 0.05, except for prostate (Supplementary Fig. 1). Prostate yielded a relatively small number of differentially spliced genes (815), despite a reasonably high number of contrasts being included (91; “Prostate1”; Supplementary Table 1).

**Fig. 3.**
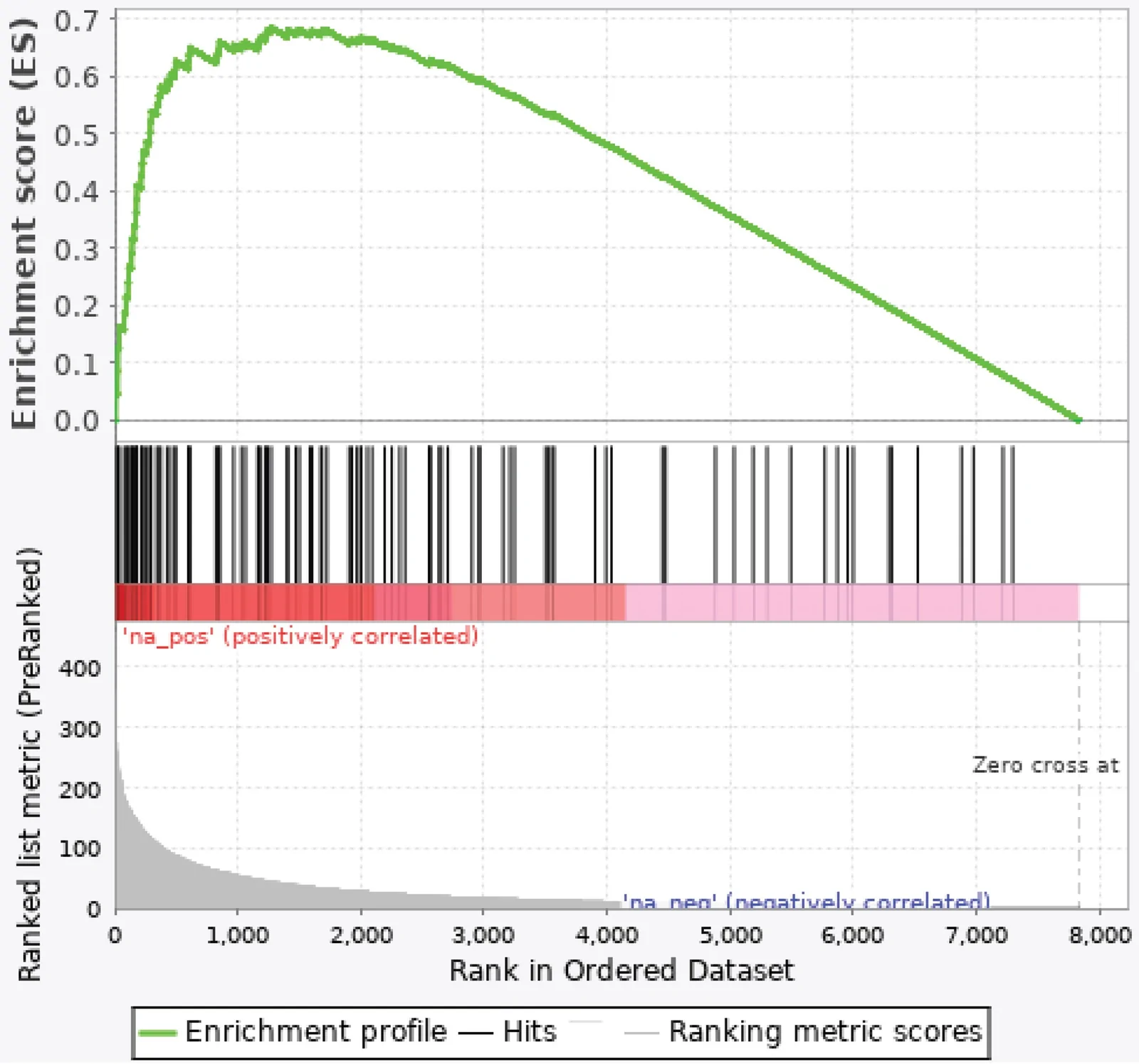
GSEA splice gene analysis. Different types of tissues from the ENCODE data were contrasted using 300 permutations (*q*-value and nominal *P*-value 9.0E−5, normalized enrichment score 1.57).

In the cancer-associated samples, the average number of genes where MAJIQ indicated differential splicing was similar to what was observed for normal tissue (Supplementary Table 2), and the enrichment analysis gave results similar to normal human tissues for about half the tumor types tested (lung adenocarcinoma, non-small cell lung cancer, colorectal cancer, and glioblastoma; Fig. 4). Other types of tumors did not show this enrichment pattern, and there was no enrichment of differential splicing of splicing components when contrasting normal adjacent tissue with lung adenocarcinomas (Fig. 4; Supplementary Table 3).

**Fig. 4.**
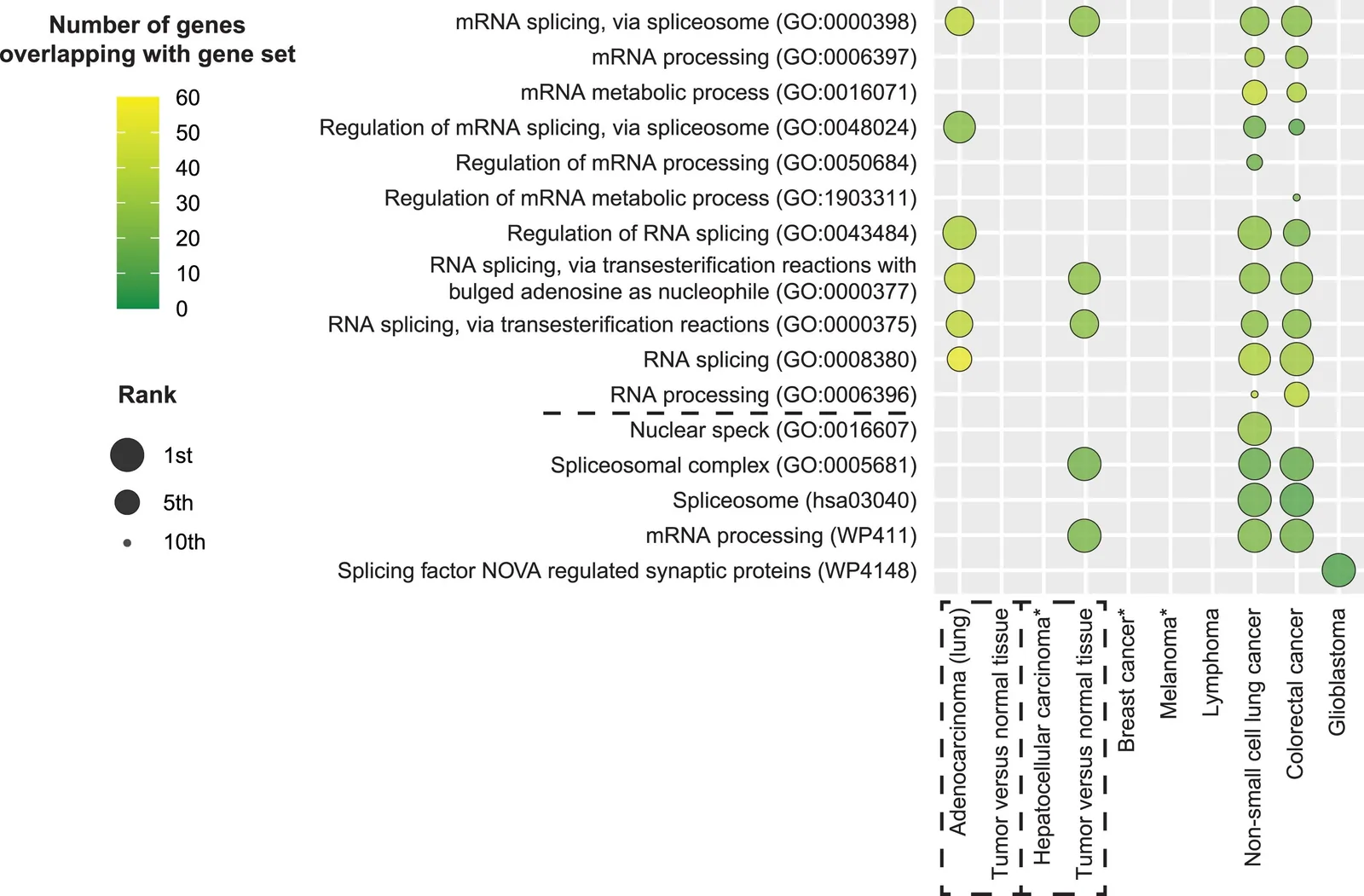
Enriched gene sets using the 20 % most frequently differentially spliced genes as input when contrasting tumors from different patients (cancer samples). Sample pairs in dotted boxes correspond to tumor vs tumor contrasts and tumor vs corresponding normal adjacent tissue from the same patients (other annotations, see Fig. 2). *No significantly enriched gene set terms.

The output from the differential splicing analysis was also processed to see if there were any differences in the differential splicing category profiles of splicing-associated genes vs other genes. Genes associated with splicing through GO terms were identified, and, in the most common differential splicing categories reported, the most pronounced differences for normal human tissue appeared to be in the alternative last exon and multiexon spanning categories of alternative splicing. Here, splicing genes seemed to be overrepresented and underrepresented, respectively (Supplementary Fig. 2b). Similar analyses using zebrafish data gave a slightly different profile (Supplementary Fig. 2a), though the number of events was a lot smaller (Supplementary Table 2).

Using only the genes associated with splicing through GO terms as the background universe, a “Molecular Function” GO term enrichment analysis of the 20 % most frequently differentially spliced splicing genes in the normal human tissue data was also performed. Only “mRNA binding” (GO:0003729) was significantly enriched within this gene set (adjusted *P* < 0.05).

## Discussion

In general, there are 3 different approaches to scoring differential splicing events (Mehmood et al. 2020). Detailed quantification of read coverage will enable the identification of exonic regions with a higher/lower coverage than expected, indicating inclusion/exclusion of transcript regions. Long-read technologies can enable accurate assembly of full-length transcripts, where noncanonical structures can readily be identified. MAJIQ (Vaquero-Garcia et al. 2023) is an example of a third category, where sequences aligned to a reference genome/transcriptome are interrogated to detect noncanonical joining of exonic regions using so-called junction reads (reads that span splice junctions). This strategy should enable sensitive detection of a wide range of alternative splicing events (alternative 5′/3′, alternative first/last exon, alternative intron, etc.) (Vaquero-Garcia et al. 2023).

MAJIQ is based on local splicing variations (LSVs) that represent splice graphs where multiple edges can come into or emerge from a reference exon. The method will allow for the detection of highly complex events that involve alternative 5′ and 3′ splice sites. A substantial fraction of alternative splicing events have been shown to be nonclassical in nature, and these would not be detected by software designed to simply score known exons as being present or absent and arranged in a specific order (Vaquero-Garcia et al. 2016). MAJIQ and other junction read-based methods will not generate full-length transcripts, and thus a set of LSVs reported from the same gene will not necessarily mean that they stem from the same transcript molecule. Regardless of approach taken, it is also not possible to systematically distinguish between mRNA molecules that are the process of a regulated cellular mechanism or the result of more stochastic events, beyond the built-in statistical tests that ensure only regions covered by a sufficient number of correctly aligned reads are considered.

The overall aim of this study was to identify genes that show differences in splice variant diversity and to investigate this using samples that were highly homologous (our original zebrafish liver data), as well as samples that can be expected to be different when it comes to gene splicing profiles (different tissue types; Fig. 3). Our approach was qualitative, as the level of expression and percent spliced-in (PSI) values were not taken into consideration when ranking the differentially spliced genes. Most software designed to detect phenomena such as differential splicing relies on contrasting samples and recording statistically significant differences. One could theoretically normalize for differences in sequencing depth and quantify the fraction of the different splice variants observed for each individual gene. A matrix of gene splice variants and samples could then be used to rank the genes according to which genes show the highest splicing variability. However, no software was identified that could do splice variant quantification using this approach in a satisfactory way, and random contrasting of samples was determined to be the best available option.

Alternative splicing can affect the phenotype of the cell (Nakka et al. 2018), or even the phenotype of the whole organism (Baker et al. 1987; Xia et al. 2024). It is believed that through evolution, complex, multicellular organisms have developed strategies for harnessing alternative splicing in order to produce all the different protein isoforms needed (Wright et al. 2022), though it has been argued that part of this could be due to decreased accuracy of splicing in higher organisms because of genetic drift and low effective population sizes (Benitiere et al. 2024). Our data indicate that there might be an evolutionary axis to the degree of differential splicing seen in splicing genes, as this tendency seems less pronounced in yeast, fruit fly, and *C. elegans* (Fig. 1). The number of differential splicing events was, however, much lower in the nonhuman samples (Supplementary Table 2), and there are also likely differences in how comprehensive the functional annotations of the genomes are in these species.

As MAJIQ is a junction read-based approach to scoring differential splicing (Vaquero-Garcia et al. 2016), any significant difference between 2 samples should imply the presence of reliably aligned junction reads that support noncanonical splicing. However, low expression levels have been linked with false positives in both exon-based and junction-based analyses (Choi et al. 2021). There will also be an inherent bias where you are more likely to detect aberrant splicing in highly expressed genes. To mitigate these limitations in functional enrichment analyses, it is recommended that only highly expressed genes are used as the background universe (Wang et al. 2024). Accordingly, the enrichment analysis was done using only the 5,000 genes that showed the highest average level of expression as background universe, though this restriction led to some tissues not getting any significantly enriched terms (see Supplementary Table 1). In order to get significant enrichment of any functional terms given the relatively low number of differential splicing events in nonhuman samples, all genes were used as background universe in these analyses.

If genes involved in splicing generally have a higher degree of expression than other genes, this might also affect the results. Using the raw counts from the STAR analysis and the 5,000 genes with the highest level of expression, genes associated with splicing seemed to have a slightly higher average level of expression, though this difference was not significant (*P* = 0.064; 2-tailed Student's *t*-test using raw count numbers) (Supplementary Fig. 3).

Another possible source of bias could be gene length/exon number, where longer genes containing more exons could have a higher number of (differential) splicing events. To check if there was an inherent bias in the splicing-associated genes, average cDNA lengths and exon numbers for genes associated with splicing through GO terms, as well as genes associated with a set of random biological process GO terms, were calculated. The splicing genes did not appear to be longer or contain more exons than other genes (Fig. 5). Instead, when looking at the complete set of genes defining the 2 sets of GO terms, splicing-associated genes were generally shorter than genes linked with random GO terms (Fig. 5).

**Fig. 5.**
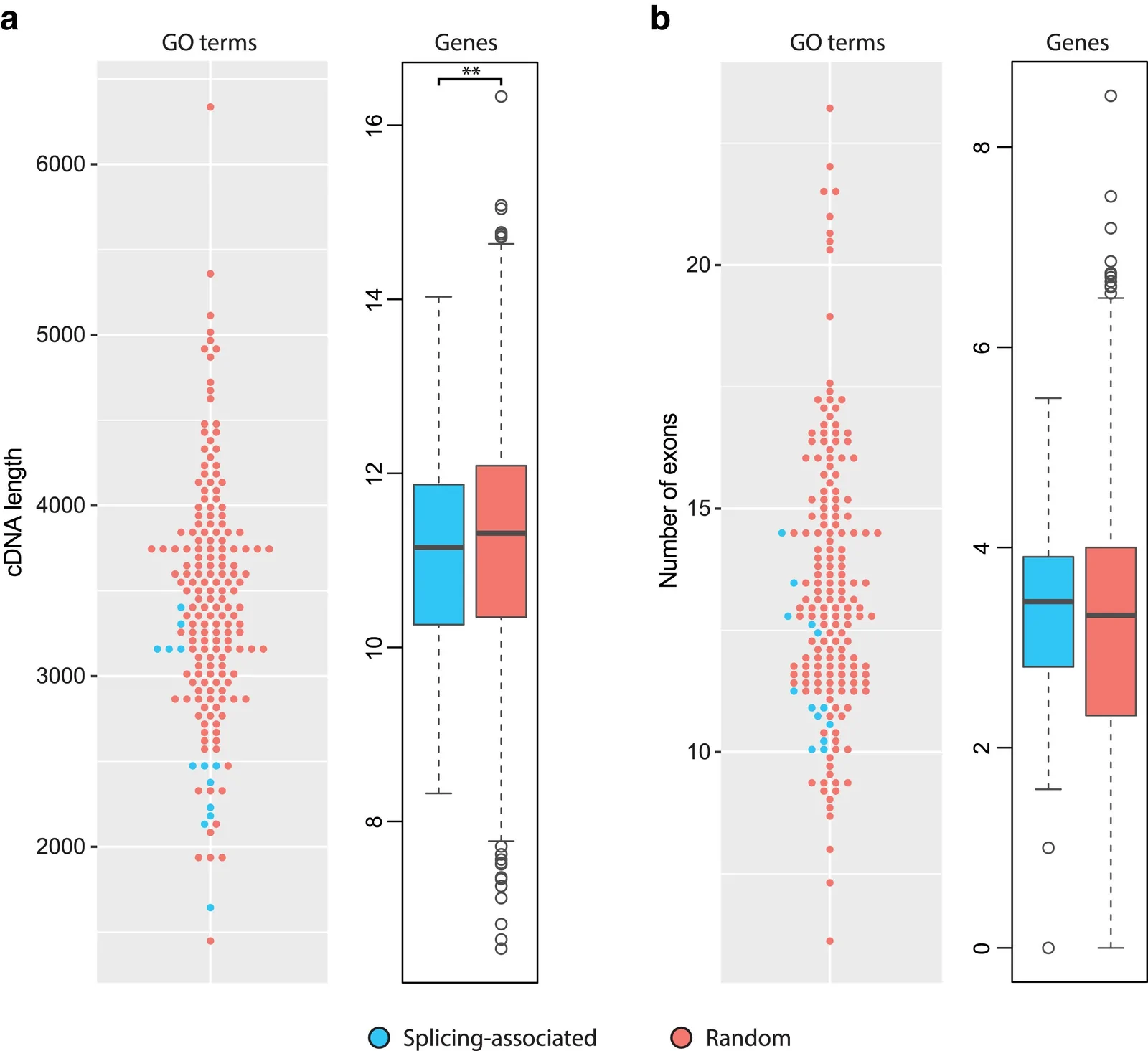
cDNA length and number of exons in genes associated with splicing GO terms vs random GO terms. GO terms connected with >20 genes related to splicing (*n* = 13) and a random set of GO terms not connected with splicing (*n* = 168) were identified, and the length of cDNA (a) and the number of exons (b) were calculated using GffRead and the R package AnnotationDbi (version 1.70.0). Bee swarm plots were made using average values for the GO term gene sets, and boxplots were made using complete lists of genes from the 2 sets (log_2_-transformed values). Significance was calculated using a 2-tailed Student's *t*-test and raw numbers.

Alternative splicing of genes expressed in skeletal muscle tissue has been investigated previously, and a highly dynamic system has been described (Nakka et al. 2018). The degree of alternative splicing has been associated with the aging process, and it has been suggested that this might be an adaptive mechanism to counter the effects of aging and adapt to different levels of fitness (Ubaida-Mohien et al. 2019a, 2019b). Interestingly, alternative splicing of splicing genes has also been shown, but seems to primarily be pronounced under specific conditions (“postexercise phosphocreatine recovery rate energetic model” (Donega et al. 2025)), which may explain the inconsistent results in our analyses (Fig. 2).

The mechanisms governing alternative splicing in the mammalian nervous system have been extensively studied (Vuong et al. 2016). The 2, primarily neuron-specific, splicing regulators NOVA1 and NOVA2 (Buckanovich et al. 1996; Yang et al. 1998; Jensen et al. 2000) have been shown to be responsible for a large number of splicing events (Ule et al. 2006), and NOVA-linked target genes were shown to be enriched in our analysis of brain-derived RNA-Seq data (Figs. 2 and 4). In addition to splicing gene terms, “Splicing factor NOVA regulated synaptic proteins” was also enriched when multiple tissue types (including different types of brain tissue) were randomly contrasted (Fig. 2).

In the umbilical cord data, there did not appear to be any enrichment of splicing-associated genes (Fig. 2). The only terms that showed enrichment were cellular compartment GO terms related to the cytoskeleton and different forms of tissue fibers (data not shown). Previous studies in mice have shown that differences in splicing patterns increase in different organs as mice mature (Mazin et al. 2021), and it is possible that the mesenchymal and epithelial stem cells present in umbilical cord tissue represent a conserved starting point preceding tissue diversification, though the number of samples included in this group was small (*n* = 6; Supplementary Table 1).

The 2 splicing genes most commonly targeted by differential splicing seem to be *HNRNPH1* and *MBNL1* (most highly ranked splicing gene in 6 and 3 different tissue types, respectively; Fig. 2). hnRNPH1 has been shown to be critical for germ cell development (Feng et al. 2022) and has been implicated in different disease states (Brownmiller and Caplen 2023), as well as being a component in drug reward systems (Yazdani et al. 2015; Fultz et al. 2021). *Muscleblind*-like paralogs have been shown to be relevant for muscle development, to be present in many isoforms, and to autoregulate their own splicing (Konieczny et al. 2018). In general, the serine/arginine-rich splicing factors (SF proteins) seemed to be the most common gene family in the list of human splicing genes with a high frequency of alternative splicing (Supplementary Table 2). These genes are also known to autoregulate their own splicing (Lareau et al. 2007; Ni et al. 2007).

Genetic lesions associated with aberrant splicing have been implemented in many different types of cancer, where both mutations affecting binding sites relevant for defining exon/intron boundaries as well as mutations linked with dysregulation of the transcription level of (aberrant) splice gene isoforms can contribute to cellular transformation (Bradley and Anczukow 2023). Given that our data indicate a high degree of differential splicing of splicing-associated genes even when contrasting the same normal tissue from different donors, it is not surprising that this trend can also be observed when contrasting tumors from different donors and tumor tissue with adjacent normal tissue from the same patient. Our results suggest that differential splicing of splicing genes could be involved in tumorigenesis, as the results from normal tissue in some cases seem to differ from tumors of the same tissue origin (Figs. 2 and 4). Tumor vs adjacent normal tissue analyses also seem to give results that are different from tumor vs tumor contrasts (Fig. 4). But several things make the interpretation complicated when analyzing data from cancer. Any mutation that increases the fitness of a cell will lead to this isoform expression profile becoming more dominant in a tumor, regardless of whether the somatic mutations driving carcinogenesis do so by affecting the splicing pattern. In addition, these data were derived from studies where different patient groups, treatment regimes, etc. were included, which could also affect the analysis.

A high diversity of splicing components may be required for the precise processing of thousands of different pre-mRNAs in a cell, including noncanonical isoforms, and a self-regulating machinery may be able to provide the cell with such a reservoir. As expected, there was a strong correlation between the overall number of differential splicing events and the number of differential splicing events in splicing-associated genes (Fig. 6a), and this trend was, to a variable extent, present in different tissue types (Fig. 6b). A possible mechanistical connection could thus be that alternatively spliced splicing components affect the splicing of other genes. An analysis was performed to investigate if differential splicing of any particular splicing-associated gene had a significant effect on the overall rate of differential splicing, and the putative helicase *EIF4A3* seemed to have the biggest impact (Fig. 6c). *EIF4A3* has, among other things, been linked with cancer development (Ye et al. 2021).

**Fig. 6.**
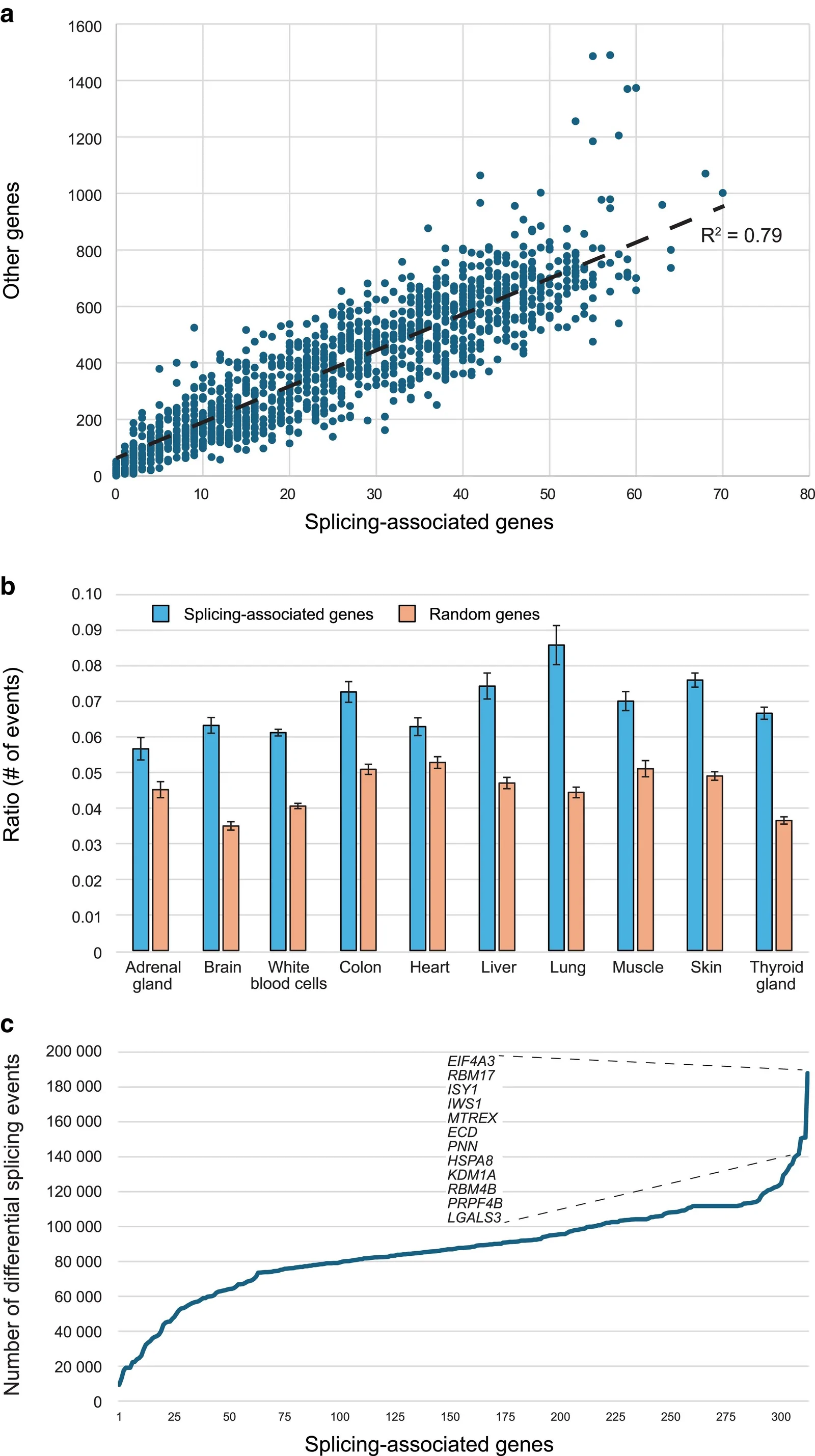
Differential splicing events in splicing genes vs other genes. (a) Scatter plot showing the total number of differential splicing events in splicing genes vs nonsplicing genes for the 10 normal human tissue types with the highest number of differential splicing events. Each data point corresponds to a contrast of 2 samples from the same tissue type, and numbers correspond to the total number of alternative splicing events reported by MAJIQ for the 2 categories of genes. (b) Ratio of the number of alternative splicing events in splicing genes vs other genes. The input data for the blue bars are the same as in (a), split according to tissue types. For the orange bars, random genes not associated with splicing were subsampled (equal number as the number of splicing-associated genes in the MAJIQ output for that particular sample contrast), and ratios of the total number of alternative splicing events were calculated. Error bars (standard deviation) indicate variability in ratios between sample contrasts. (c) Impact of differential splicing of splicing-associated genes on overall number of differential splicing events. For all random contrasts using normal human tissue samples, lists with (overlapping) sets of contrasts corresponding to which of the splicing genes (*n* = 313 after removal of genes differentially spliced in <5 contrasts) that showed differential splicing were generated. Each list was coupled with the number of differential splicing events recorded for that particular contrast. The average number of differential splicing events per splicing gene was calculated, sorted, and plotted. A series of splicing-associated genes that correlated with sudden increases in the average number of events to the extreme right of the diagram has been indicated.

As has been suggested for SF genes, it is also possible that the creation of alternatively spliced transcripts targeted for nonsense-mediated decay might help to precisely regulate the number of functional transcripts in a cell (Jumaa and Nielsen 1997; Ni et al. 2007).

It has been suggested that the high degree of alternative splicing in more complex organisms may largely be due to splicing errors, especially when looking at low-abundance isoforms (Benitiere et al. 2024). However, studies have also shown that the majority of alternatively spliced transcripts are translated (Weatheritt et al. 2016), implying that they are functional. Whether or not all the splice variants detected in our data are biologically relevant is unclear, and it is possible that some of the aberrant junction reads detected are not the product of tightly regulated processes, in spite of the statistical filtering done by MAJIQ. Detailed output from LSVs scored as being changed between samples showed average PSI values to be around 0.6 (Supplementary Table 6). Assuming reasonable coverage of the canonical form of the transcripts, this number suggests that many differential LSVs were supported by a fair number of reads. Many complex sets of differential splicing events were also repeatedly detected (Supplementary Table 5). Our analyses showed a good degree of reproducibility when comparing results from the same tissue types using data from different sources (zebrafish liver; Fig. 1, and human skin; Fig. 2), except for skeletal muscle (see discussion above). An aspect of our approach that could lead to an underestimation of the splicing dynamics is the focus on differentially spliced transcripts. If, for instance, a gene has an equally high degree of alternative splicing in 2 samples, contrasting them will not lead to this event being scored as a differential splicing event.

Alternative splicing of specific splicing-associated genes has been observed in several studies previously, including studies on neuronal differentiation and signaling (Lee et al. 2009; Fiszbein et al. 2016), erythropoiesis (Yamamoto et al. 2009), and muscle adaptation (Donega et al. 2025). A likely important contributor to an overall trend of shifting splicing patterns for genes associated with splicing is the autoregulating SF proteins (Jumaa and Nielsen 1997; Ni et al. 2007). Modeling of splicing has also revealed a high degree of cross-regulation (Kosti et al. 2012), and in some smaller datasets, general alternative splicing of splicing genes has been indicated (Mehmood et al. 2020; Donega et al. 2025). Most of these studies have, however, focused on samples derived from different tissues, developmental stages, or metabolic states, or defined by other factors thought to be causally linked with the splicing signatures observed. We have included samples that are highly homologous but still observe significant differences in multiple tissue types and species. Whether this reflects an underlying, highly dynamic diversity within the specimens or more fundamental differences between the sampled individuals remains unclear.

## Supplementary Material

jkag213_Supplementary_Data

## Data Availability

RNA-Seq data were submitted to the NCBI SRA database as BioProject PRJNA1237738. Supplemental material available at *G3* online.
