# Supplementary material for: Splicing genes as main targets of differential splicing
Source: G3 (Bethesda). 2026 Aug 14;16(10):jkag213. doi: 10.1093/g3journal/jkag213 (PMC13643759; doi:10.1093/g3journal/jkag213)
Supplement: Supplementary file 1 [file jkag213_supplementary_data.zip › Supplementary_Figure_1_G3-2026-406854.pdf]

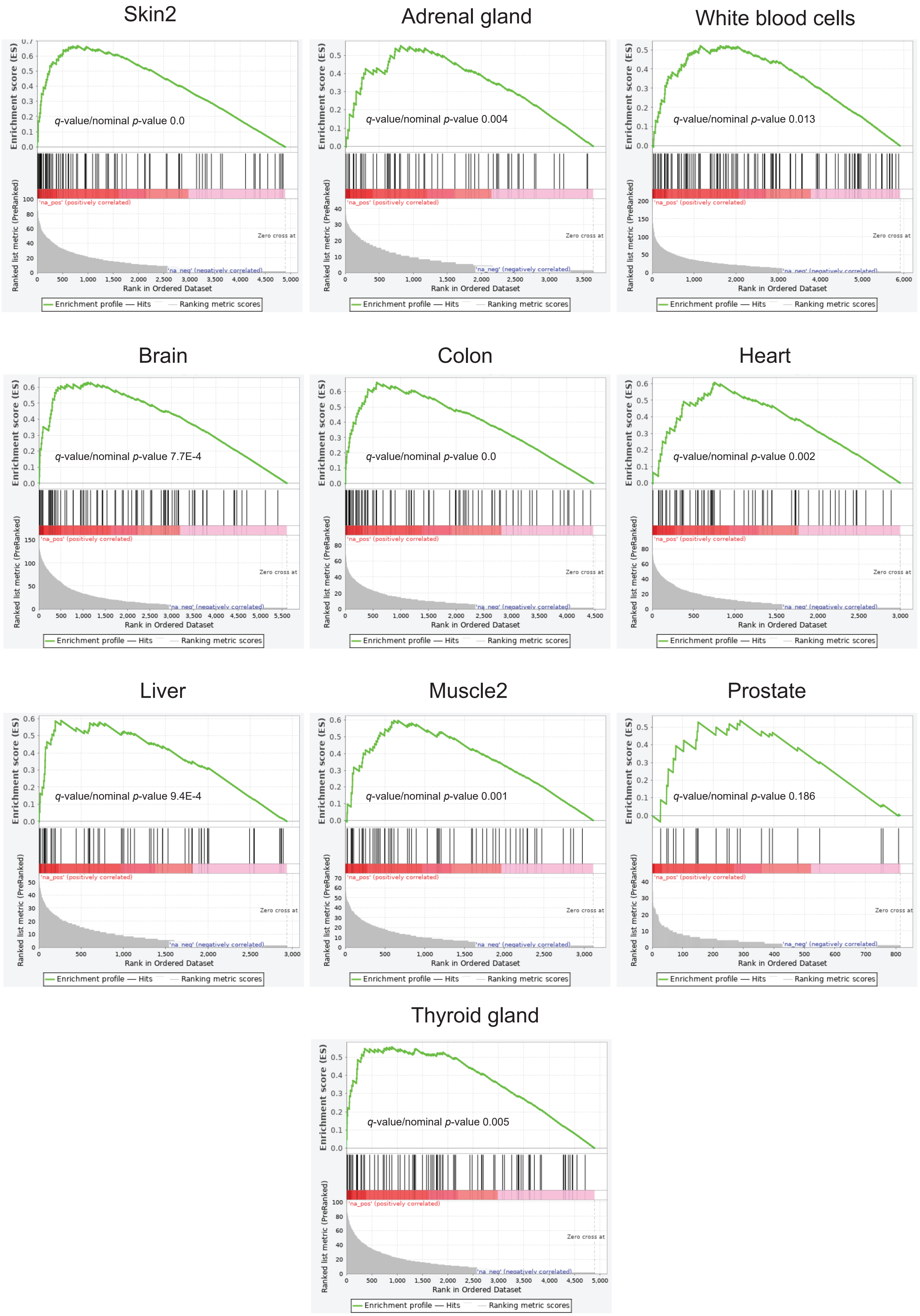
Skin2
q-value/nominal p-value 0.0
Adrenal gland
q-value/nominal p-value 0.004
White blood cells
q-value/nominal p-value 0.013
Brain
q-value/nominal p-value 7.7E-4
Colon
q-value/nominal p-value 0.0
Heart
q-value/nominal p-value 0.002
Liver
q-value/nominal p-value 9.4E-4
Muscle2
q-value/nominal p-value 0.001
Prostate
q-value/nominal p-value 0.186
Thyroid gland
q-value/nominal p-value 0.005
Enrichment score (ES)
Ranked list metric (PreRanked)
Rank in Ordered Dataset
'na_pos' (positively correlated)
'na_neg' (negatively correlated)
Zero cross at
Enrichment profile — Hits
Ranking metric scores
