# Supplementary material for: Splicing genes as main targets of differential splicing
Source: G3 (Bethesda). 2026 Aug 14;16(10):jkag213. doi: 10.1093/g3journal/jkag213 (PMC13643759; doi:10.1093/g3journal/jkag213)
Supplement: Supplementary file 1 [file jkag213_supplementary_data.zip › Supplementary_Figure_2_G3-2026-406854.pdf]

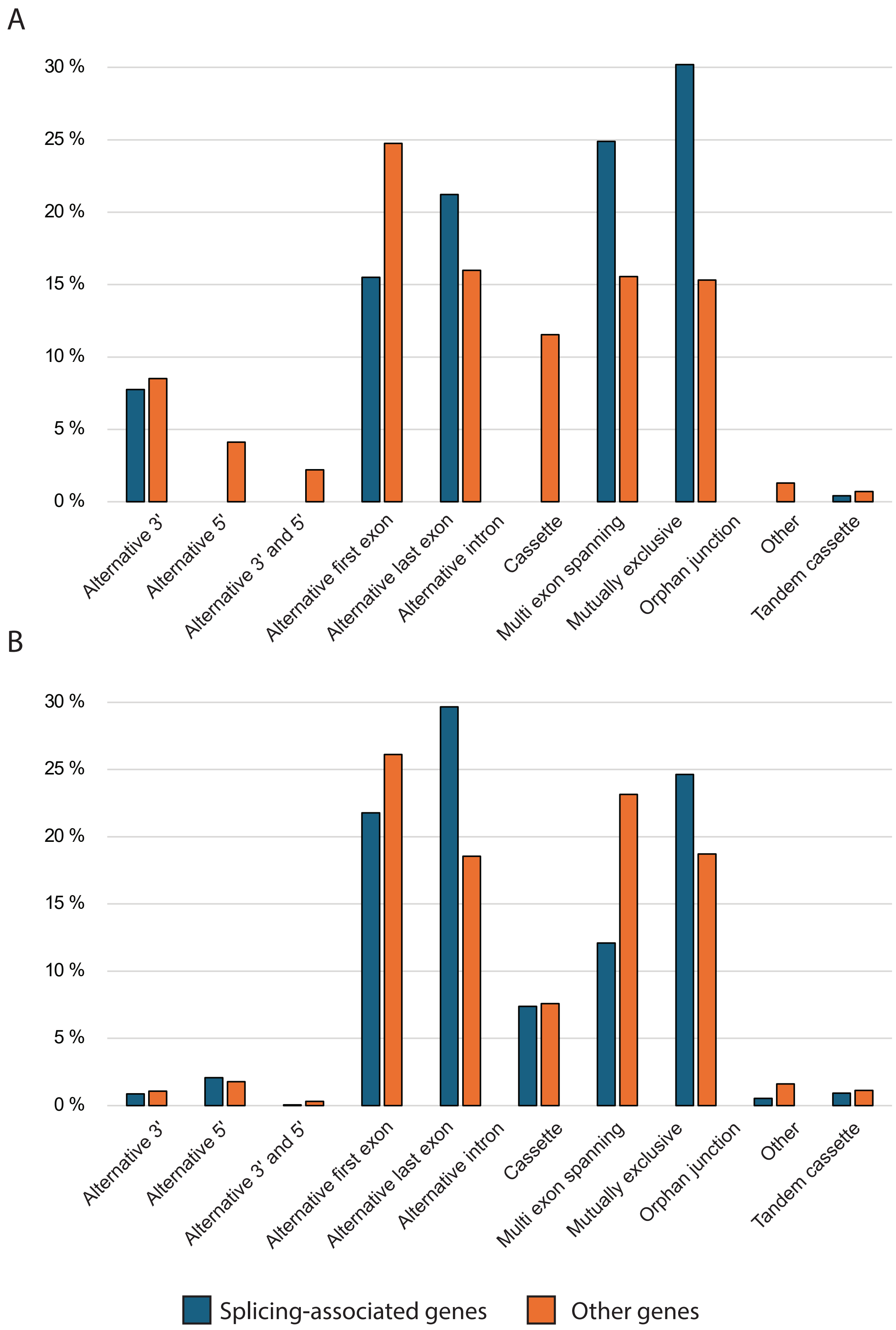
A
30 %
25 %
20 %
15 %
10 %
5 %
0 %
Alternative 3'
Alternative 5'
Alternative 3' and 5'
Alternative first exon
Alternative last exon
Alternative intron
Cassette
Multi exon spanning
Mutually exclusive
Orphan junction
Other
Tandem cassette
B
30 %
25 %
20 %
15 %
10 %
5 %
0 %
Alternative 3'
Alternative 5'
Alternative 3' and 5'
Alternative first exon
Alternative last exon
Alternative intron
Cassette
Multi exon spanning
Mutually exclusive
Orphan junction
Other
Tandem cassette
Splicing-associated genes
Other genes
