# Supplementary material for: Splicing genes as main targets of differential splicing
Source: G3 (Bethesda). 2026 Aug 14;16(10):jkag213. doi: 10.1093/g3journal/jkag213 (PMC13643759; doi:10.1093/g3journal/jkag213)
Supplement: Supplementary file 1 [file jkag213_supplementary_data.zip › Supplementary_Figure_3_G3-2026-406854.pdf]

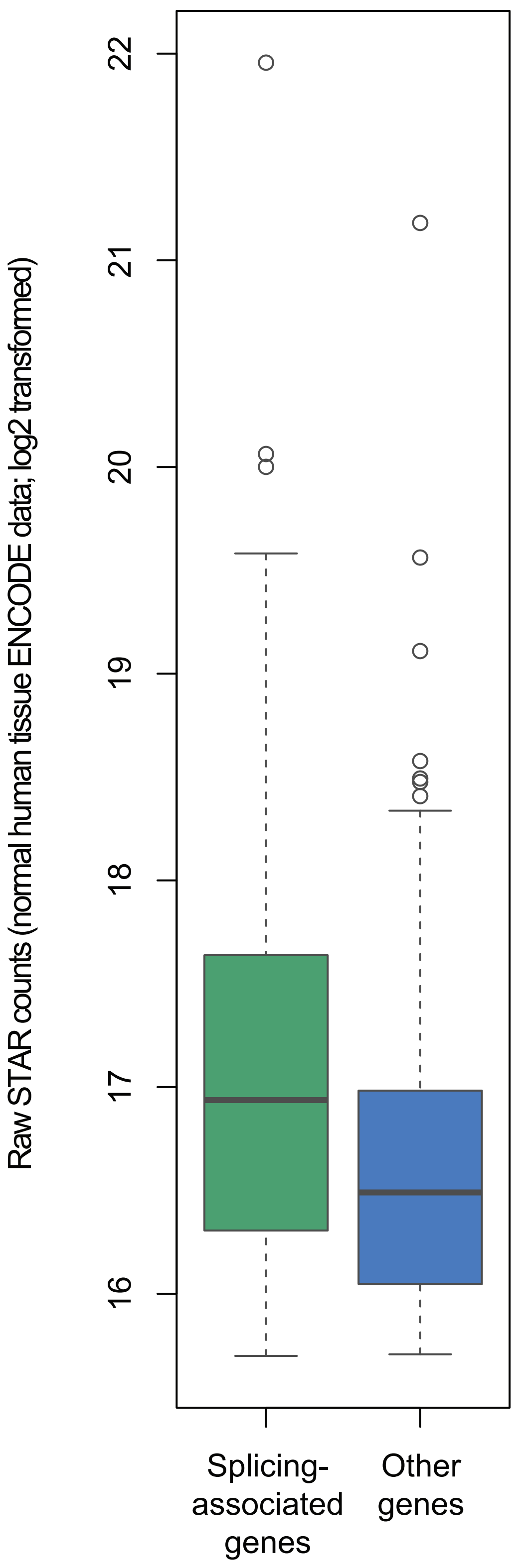
Raw STAR counts (normal human tissue ENCODE data; log2 transformed)
22
21
20
19
18
17
16
Splicing-associated genes
Other genes
