# Supplementary material for: Splicing genes as main targets of differential splicing
Source: G3 (Bethesda). 2026 Aug 14;16(10):jkag213. doi: 10.1093/g3journal/jkag213 (PMC13643759; doi:10.1093/g3journal/jkag213)
Supplement: Supplementary file 1 [file jkag213_supplementary_data.zip › Supplementary_Figure_Legends_G3-2026-406854.docx]

Supplementary figure 1. GSEA spliceosome analysis of all normal human tissues with > 50 contrasts.

Supplementary figure 2. Frequency of different types of local splicing variations (LSVs) scored by MAJIQ. Only LSVs with probability of changing = 1 in voila modulize output were used (default parameters; junctions.tsv output). Genes were scored as being associated with splicing using Gene Ontology Biological Process terms. A) Original zebrafish liver data and B) human ENCODE data from random contrasts of different tissue types.

Supplementary figure 3. Comparison of expression level of splicing-associated genes versus other genes in gene set used for functional enrichment analysis.
