# Supplementary material for: Splicing genes as main targets of differential splicing
Source: G3 (Bethesda). 2026 Aug 14;16(10):jkag213. doi: 10.1093/g3journal/jkag213 (PMC13643759; doi:10.1093/g3journal/jkag213)
Supplement: Supplementary file 1 [file jkag213_supplementary_data.zip › Supplementary_Table_1_G3-2026-406854.docx]

Supplementary table 1. Samples included in the study.

| **Tissue/label** | **Contrasts** | **BioProject** | **Genome (Ensemble 113)** |
| --- | --- | --- | --- |
| Yeast | 300 | PRJNA778214 (Kaya et al. 2021) | Saccharomyces_cerevisiae.R64-1-1 |
| Mouse | 300 | PRJNA747753 (Dahl et al. 2021) | Mus_musculus.GRCm39 |
| Random tissue contrasts | 300 | PRJNA30709 (Consortium 2012) | Homo_sapiens.GRCh38 |
| Adenocarcinoma (lung) | 300 | PRJEB2784 (Ju et al. 2012) | Homo_sapiens.GRCh38 |
| Hepatocellular carcinoma | 300 | PRJNA310012 (Liu et al. 2016) | Homo_sapiens.GRCh38 |
| Breast cancer | 300 | PRJNA586892 (Hugh et al. 2022) | Homo_sapiens.GRCh38 |
| Melanoma | 300 | PRJNA715643 (Plaschka et al. 2022) | Homo_sapiens.GRCh38 |
| Non-small cell lung cancer | 300 | PRJNA786565 (Limagne et al. 2022) | Homo_sapiens.GRCh38 |
| Glioblastoma | 300 | PRJNA998933 (Lemarie et al. 2023) | Homo_sapiens.GRCh38 |
| Muscle2 | 300 | PRJNA1021259 (Petersen et al. 2024) | Homo_sapiens.GRCh38 |
| Skin2 | 300 | PRJNA481993 (Ahn et al. 2018) | Homo_sapiens.GRCh38 |
| Lymphoma | 298 | PRJNA727804 (Plaks et al. 2021) | Homo_sapiens.GRCh38 |
| Colorectal cancer | 284 | PRJNA988034 (Thibaudin et al. 2023) | Homo_sapiens.GRCh38 |
| White blood cells* | 253 | PRJNA1163973 | Homo_sapiens.GRCh38 |
| Brain | 231 | PRJNA30709 (Consortium 2012) | Homo_sapiens.GRCh38 |
| Zebrafish | 171 | PRJNA911635 (Leonard et al. 2023) | Danio_rerio.GRCz11 |
| Skin1 | 136 | PRJNA30709 (Consortium 2012) | Homo_sapiens.GRCh38 |
| Thyroid gland | 136 | PRJNA30709 (Consortium 2012) | Homo_sapiens.GRCh38 |
| Colon | 105 | PRJNA30709 (Consortium 2012) | Homo_sapiens.GRCh38 |
| Heart | 105 | PRJNA30709 (Consortium 2012) | Homo_sapiens.GRCh38 |
| Prostate1 | 91 | PRJNA209978 (Rajan et al. 2014) | Homo_sapiens.GRCh38 |
| *C. elegans* | 91 | PRJNA33023 (Hillier et al. 2009) | Caenorhabditis_elegans.WBcel235 |
| Liver | 78 | PRJNA30709 (Consortium 2012) | Homo_sapiens.GRCh38 |
| Muscle1 | 78 | PRJNA30709 (Consortium 2012) | Homo_sapiens.GRCh38 |
| Tumor versus normal tissue (lung) | 69 | PRJEB2784 (Ju et al. 2012) | Homo_sapiens.GRCh38 |
| Fruit fly (carcass) | 66 | PRJEB22205 (Krause et al. 2022) | Drosophila_melanogaster.BDGP6.46 |
| Adrenal gland* | 55 | PRJNA30709 (Consortium 2012) | Homo_sapiens.GRCh38 |
| Tumor versus normal tissue (liver) | 50 | PRJNA310012 (Liu et al. 2016) | Homo_sapiens.GRCh38 |
| Zebrafish (original data) | 45 | PRJNA1237738 | Danio_rerio.GRCz11 |
| Lung | 45 | PRJNA30709 (Consortium 2012) | Homo_sapiens.GRCh38 |
| Stomach | 45 | PRJNA30709 (Consortium 2012) | Homo_sapiens.GRCh38 |
| Spleen | 45 | PRJNA30709 (Consortium 2012) | Homo_sapiens.GRCh38 |
| Fruit fly (hindgut) | 36 | PRJEB22205 (Krause et al. 2022) | Drosophila_melanogaster.BDGP6.46 |
| Fruit fly (midgut) | 36 | PRJEB22205 (Krause et al. 2022) | Drosophila_melanogaster.BDGP6.46 |
| Esophagus squamous epithelium* | 28 | PRJNA30709 (Consortium 2012) | Homo_sapiens.GRCh38 |
| Esophagus muscularis mucosa* | 28 | PRJNA30709 (Consortium 2012) | Homo_sapiens.GRCh38 |
| Gastroesophageal_sphincter | 28 | PRJNA30709 (Consortium 2012) | Homo_sapiens.GRCh38 |
| Testis* | 28 | PRJNA30709 (Consortium 2012) | Homo_sapiens.GRCh38 |
| Omental fat pad | 21 | PRJNA30709 (Consortium 2012) | Homo_sapiens.GRCh38 |
| Uterus | 21 | PRJNA30709 (Consortium 2012) | Homo_sapiens.GRCh38 |
| Aorta | 15 | PRJNA30709 (Consortium 2012) | Homo_sapiens.GRCh38 |
| Adipose tissue | 15 | PRJNA30709 (Consortium 2012) | Homo_sapiens.GRCh38 |
| Kidney | 15 | PRJNA30709 (Consortium 2012) | Homo_sapiens.GRCh38 |
| Tibial nerve* | 15 | PRJNA30709 (Consortium 2012) | Homo_sapiens.GRCh38 |
| Peyer's patch* | 15 | PRJNA30709 (Consortium 2012) | Homo_sapiens.GRCh38 |
| Pancreas | 10 | PRJNA30709 (Consortium 2012) | Homo_sapiens.GRCh38 |
| Breast epithelium | 6 | PRJNA30709 (Consortium 2012) | Homo_sapiens.GRCh38 |
| Prostate2 | 6 | PRJNA30709 (Consortium 2012) | Homo_sapiens.GRCh38 |
| Tongue* | 6 | PRJNA30709 (Consortium 2012) | Homo_sapiens.GRCh38 |
| Ovary | 6 | PRJNA30709 (Consortium 2012) | Homo_sapiens.GRCh38 |
| Spinal cord | 6 | PRJNA30709 (Consortium 2012) | Homo_sapiens.GRCh38 |
| Umbilical cord | 6 | PRJNA30709 (Consortium 2012) | Homo_sapiens.GRCh38 |
| Urinary bladder | 6 | PRJNA30709 (Consortium 2012) | Homo_sapiens.GRCh38 |
| Camera-type eye | 3 | PRJNA30709 (Consortium 2012) | Homo_sapiens.GRCh38 |

* - No significantly enriched terms found by clusterProfiler.

**REFERENCES**

Ahn, R., D. Yan, H.W. Chang, K. Lee, S. Bhattarai *et al.*, 2018 RNA-seq and flow-cytometry of conventional, scalp, and palmoplantar psoriasis reveal shared and distinct molecular pathways. *Sci Rep* 8 (1):11368.

Consortium, E.P., 2012 An integrated encyclopedia of DNA elements in the human genome. *Nature* 489 (7414):57-74.

Dahl, H., D.M. Eide, T. Tengs, N. Duale, J.H. Kamstra *et al.*, 2021 Perturbed transcriptional profiles after chronic low dose rate radiation in mice. *PLoS One* 16 (8):e0256667.

Hillier, L.W., V. Reinke, P. Green, M. Hirst, M.A. Marra *et al.*, 2009 Massively parallel sequencing of the polyadenylated transcriptome of C. elegans. *Genome Research* 19 (4):657-666.

Hugh, J.C., L.S.J. Haddon, J.M. Githaka, G. Bigras, X. Hu *et al.*, 2022 DREAM, a possible answer to the estrogen paradox of the Women's Health Initiative Trial. *Heliyon* 8 (1):e08666.

Ju, Y.S., W.C. Lee, J.Y. Shin, S. Lee, T. Bleazard *et al.*, 2012 A transforming KIF5B and RET gene fusion in lung adenocarcinoma revealed from whole-genome and transcriptome sequencing. *Genome Res* 22 (3):436-445.

Kaya, A., C.Z.J. Phua, M. Lee, L. Wang, A. Tyshkovskiy *et al.*, 2021 Evolution of natural lifespan variation and molecular strategies of extended lifespan in yeast. *Elife* 10.

Krause, S.A., G. Overend, J.A.T. Dow, and D.P. Leader, 2022 FlyAtlas 2 in 2022: enhancements to the Drosophila melanogaster expression atlas. *Nucleic Acids Res* 50 (D1):D1010-D1015.

Lemarie, A., V. Lubrano, C. Delmas, A. Lusque, J.P. Cerapio *et al.*, 2023 The STEMRI trial: Magnetic resonance spectroscopy imaging can define tumor areas enriched in glioblastoma stem-like cells. *Sci Adv* 9 (44):eadi0114.

Leonard, E.R., E.S. Marques, M.A. Roy, S.M. Conlin, R. Ranjan *et al.*, 2023 Dietary exposure to the food preservative tert-Butylhydroquinone (tBHQ) impairs zebrafish (Danio rerio) survival, growth, organ development, and gene expression in Nrf2a-dependent and independent ways. *Food Chem Toxicol* 176:113788.

Limagne, E., L. Nuttin, M. Thibaudin, E. Jacquin, R. Aucagne *et al.*, 2022 MEK inhibition overcomes chemoimmunotherapy resistance by inducing CXCL10 in cancer cells. *Cancer Cell* 40 (2):136-152 e112.

Liu, G., G. Hou, L. Li, Y. Li, W. Zhou *et al.*, 2016 Potential diagnostic and prognostic marker dimethylglycine dehydrogenase (DMGDH) suppresses hepatocellular carcinoma metastasis in vitro and in vivo. *Oncotarget* 7 (22):32607-32616.

Petersen, M.C., G.I. Smith, H.H. Palacios, S.S. Farabi, M. Yoshino *et al.*, 2024 Cardiometabolic characteristics of people with metabolically healthy and unhealthy obesity. *Cell Metab* 36 (4):745-761 e745.

Plaks, V., J.M. Rossi, J. Chou, L. Wang, S. Poddar *et al.*, 2021 CD19 target evasion as a mechanism of relapse in large B-cell lymphoma treated with axicabtagene ciloleucel. *Blood* 138 (12):1081-1085.

Plaschka, M., V. Benboubker, M. Grimont, J. Berthet, L. Tonon *et al.*, 2022 ZEB1 transcription factor promotes immune escape in melanoma. *J Immunother Cancer* 10 (3).

Rajan, P., I.M. Sudbery, M.E. Villasevil, E. Mui, J. Fleming *et al.*, 2014 Next-generation sequencing of advanced prostate cancer treated with androgen-deprivation therapy. *Eur Urol* 66 (1):32-39.

Thibaudin, M., J.D. Fumet, B. Chibaudel, J. Bennouna, C. Borg *et al.*, 2023 First-line durvalumab and tremelimumab with chemotherapy in RAS-mutated metastatic colorectal cancer: a phase 1b/2 trial. *Nat Med* 29 (8):2087-2098.
