# Supplementary material for: Splicing genes as main targets of differential splicing
Source: G3 (Bethesda). 2026 Aug 14;16(10):jkag213. doi: 10.1093/g3journal/jkag213 (PMC13643759; doi:10.1093/g3journal/jkag213)
Supplement: Supplementary file 1 [file jkag213_supplementary_data.zip › Supplementary_Table_5_G3-2026-406854.docx]

Supplementary table 5. Most common differential splicing events reported for splicing genes (MAJIQ analysis; normal human tissue ENCODE data; all contrast groups).

| **Gene** | **Event (MAJIQ annotation)** | **Count** |
| --- | --- | --- |
| *CLK1* | cassette^1\|ir^5\|multi_exon_spanning^2 | 175 |
| *HNRNPH1* | cassette^1\|ir^5\|multi_exon_spanning^2 | 126 |
| *HNRNPH1* | ir^1 | 119 |
| *HNRNPH1* | ir^2 | 112 |
| *HNRNPLL* | ir^1 | 109 |
| *QKI* | alt3^1\|putative_alt3^2\|ir^5 | 92 |
| *HNRNPL* | afe^1 | 91 |
| *HNRNPA2B1* | cassette^1\|ir^5\|multi_exon_spanning^2 | 90 |
| *HNRNPK* | alt5^1\|afe^2\|other^1 | 87 |
| *PRPF40A* | cassette^1\|ir^2 | 87 |
| *WTAP* | afe^1 | 80 |
| *QKI* | ir^2 | 73 |
| *SRSF6* | cassette^1\|ir^2 | 73 |
| *ZRANB2* | ir^2\|ale^2 | 72 |
| *SRSF7* | cassette^1\|ir^4 | 69 |
| *SRSF1* | ir^2\|putative_afe^2 | 69 |
| *SF1* | cassette^1\|ir^5\|multi_exon_spanning^2 | 67 |
| *KDM1A* | cassette^1\|ir^2 | 66 |
| *TIA1* | cassette^2\|tandem_cassette^1\|putative_alt5^1\|ir^7\|multi_exon_spanning^4 | 65 |
| *SRSF5* | putative_alt3^1\|ir^3\|multi_exon_spanning^2 | 64 |
| *HNRNPA3* | alt5^1\|ir^2\|afe^2 | 64 |
| *RBFOX2* | afe^1 | 62 |
| *SRSF5* | ir^2\|multi_exon_spanning^1 | 62 |
| *HNRNPA3* | alt5^1 | 62 |
| *ZRANB2* | ir^1 | 61 |
| *SRSF10* | ir^2\|afe^2\|multi_exon_spanning^1 | 61 |
| *CLK1* | ir^1 | 60 |
| *SF3B1* | putative_alt5^1\|ir^3 | 60 |
| *HNRNPA2B1* | ir^1 | 60 |
| *CCNL1* | cassette^2\|alt5^1\|ir^7\|multi_exon_spanning^2 | 59 |
| *FXR1* | cassette^2\|tandem_cassette^1\|putative_alt3^1\|ir^7\|multi_exon_spanning^4 | 58 |
| *RBM39* | cassette^1\|ir^5\|ale^1\|afe^2\|multi_exon_spanning^3 | 58 |
| *ARL6IP4* | afe^1 | 56 |
| *SRSF6* | cassette^1\|ir^3\|afe^1\|multi_exon_spanning^2 | 55 |
| *HNRNPU* | putative_alt5^1\|ir^1 | 53 |
| *LUC7L* | cassette^1\|ir^3\|afe^1\|multi_exon_spanning^2 | 51 |
| *RBM5* | cassette^1\|ir^4 | 50 |
| *AKAP8L* | cassette^1\|ir^4 | 50 |
| *MBNL1* | cassette^1\|ir^4 | 49 |
| *PPIL1* | afe^1 | 46 |
| *SRSF5* | alt5^3\|ir^6 | 45 |
| *PPIG* | alt5^1\|afe^2\|other^1 | 44 |
| *GRSF1* | alt5^1\|afe^2 | 43 |
| *NONO* | cassette^1\|ale^1\|multi_exon_spanning^1\|other^1 | 43 |
| *CLK4* | ir^2\|ale^2 | 42 |
| *PUF60* | cassette^1 | 42 |
| *SRSF5* | ir^1 | 40 |
| *HNRNPK* | alt5^1\|afe^7\|multi_exon_spanning^1\|other^1 | 40 |
| *HNRNPH1* | ir^3\|putative_afe^1 | 40 |
| *ACIN1* | cassette^1 | 38 |
